# Culture and mood disorders: the effect of abstraction in image, narrative and film on depression and anxiety

**DOI:** 10.1136/medhum-2018-011459

**Published:** 2019-10-31

**Authors:** James Carney

**Affiliations:** Arts & Humanities/Centre for Culture and Evolution, Brunel University London, Uxbridge UB8 3PH, UK

**Keywords:** Medical Humanities, Narrative Medicine, Psychotherapy, Art, Film

## Abstract

Can cultural representations be used to therapeutic effect in the treatment of mood disorders like depression and anxiety? This article develops a theoretical framework that outlines how this might be achieved by way of mid-level cultural metrics that allow otherwise heterogeneous forms of representation to be grouped together. Its prediction is that abstract representations—as measured by Shannon entropy—will impact positively on anxiety, where concrete representations will positively impact on depression. The background to the prediction comes from construal level theory, a branch of social psychology that deals with the effects of abstraction on psychological distance; the types of cultural representations analysed include image, narrative and film. With a view to evaluating the hypothesis, the article surveys the empirical literature in art therapy, creative bibliotherapy and cinema therapy.

## Introduction

How does culture relate to mental health? If this question is easy to ask, it is less easily answered. Both culture and mental health are highly contested terms, meaning that no two interlocutors are likely to agree on even basic definitions. In the first instance, this is because culture comprises categories of assortment like ethnicity, nationality and class, as well as symbolic practices such as religion, art and sport—a scenario that disperses the consideration of culture and mental health across a range of objects and disciplines.1 In the second, the fraught political history of medical classification means that mental pathologies are the site of some of the most vigorous debates in the critical social sciences, ensuring that no one account of what constitutes mental illness attracts majority assent. While such tensions have been productive when it comes to unsettling normative diagnostic labels,2 there can be no doubt that they have also had the effect of balkanising the literature. To be sure, there is no shortage of interesting results and discussions.3 Nevertheless, it is equally true that the lack of a background theoretical framework militates against a deeper understanding of how culture shapes (and is shaped by) the experience of mental illness.

As no single exposition is likely to successfully anticipate what such a framework will look like in totality, I shall not attempt to deliver one here. Instead, I hope to outline how one metric of variation—abstraction—promises to connect different modes of cultural representation and mood disorders in a way relevant to thinking about how cultural objects might be exploited therapeutically. The background to this will come from recent work in construal level theory (CLT), a branch of social psychology that links the experience of psychological distance to the experience of abstraction. The animating claim of CLT is that the experience of abstraction cues expectations of psychological distance in the spatial, temporal, probabilistic and social spheres, just as the experience of distance cues expectations of abstraction.4 In itself, this observation seems relatively modest, but the achievement of CLT is to identify in abstraction a mid-level metric that is sufficiently general to be detectable across a wide range of stimuli, yet concrete enough to say something non-trivial about any given one of these. What this means in practice is that CLT allows for connection to be made between the experience of cultural artefacts in the broad sense and the disposition to behave in certain ways. That is, if the experience of abstraction (or concreteness) can be linked to expanded or contracted mental horizons, then the vehicle of that experience can be identified as a stimulus for the actions that take place within those horizons. And this, indeed, is what the literature shows: the experimental programme of CLT provides many examples linking moral, social, practical and creative behaviours (among others) to the experiences of abstraction and concreteness.5

Where these discoveries connect with the mood disorders is by way of a separate body of work that identifies depression and anxiety to be problems of overgeneralisation and undergeneralisation. While both conditions are obviously more complicated than can be explained by a one-dimensional characterisation such as this, it remains the case that it picks out an important component of variation. Depression, for instance, is typically experienced as ‘a feeling of being dislodged from everyday activity’, 6 leaving the sufferer ‘an isolated object in a world without relationships’.7 Conversely, anxiety generates the sense of ‘being too much in contact with reality’8 and ‘the fearful anticipation of a catastrophe one is hopeless to prevent’. 9 What is visible in both conditions is attentional capture by interpretive frames that either strip away or exaggerate the constituent details of ordinary human experience. To this extent, CLT provides an empirically sanctioned bridge between modes of cultural expression (and the historical factors behind them) and the experience of mental illness. It hardly needs stating that this connection may well allow for insights into how cultural representations can be most effectively leveraged in support of therapeutic interventions.

Starting from these observations, I shall proceed here by delivering an overview of how different modes of cultural expression can evince abstraction and concreteness, and commenting on the relevance of this for anxiety and depression. For the most part, the cultural forms I will discuss relate to image, narrative and film. (Music I shall not consider due to lack of specialist knowledge.) All these genres of representation use different materials against different horizons of expectation, with the result that there is no simple sense in which they can be said to be abstract or concrete. Nevertheless, if the ideas I propose here are to be tested, defensible and reliable measures of this representational tendency need to be developed. Necessarily, doing this will involve a wide-ranging discussion of cultural representation; to no less a degree, it is unavoidable that some of these discussions must deal with technical concepts in linguistics, information theory and cognitive science. However, to develop even one metric of health-relevant variation that extends across several cultural forms and which admits of objective estimation must surely be admitted as a useful exercise. At the very least, it challenges the de facto approach, which is to treat different modes of expression on an individual basis, without ever taking a global overview.

## CLT, depression and anxiety

If CLT and mood disorders are to contextualise an analysis of cultural forms, then the relation between the two needs to be properly explicated. The present section will do this by way of a short review of the CLT literature that discusses how its findings might be brought into contact with cognate findings on depression and anxiety. This will deliver the background knowledge needed for the subsequent engagement with specific modes of cultural representation.

For all that CLT is communicated in a specialist vocabulary, the insights behind it are rather intuitive. Central among these is the idea that human cognition persistently links abstraction and distance. ‘Abstraction’ in this connection relates to the level of detail associated with a stimulus, with CLT proponents labelling a high-detail stimulus as having a ‘low’ construal level and a low-detail stimulus as having a ‘high’ construal level. ‘Distance’ equates with psychological distance, which comprises distance in the spatial, temporal, probabilistic and social dimensions. (The adjective ‘psychological’ denotes that it is the subject’s perception or imagination of distance that matters, rather than any physical measure of distance, even if the two are frequently connected.) The CLT proposal is that that expectations of distance on these four dimensions are cued by high-construal level stimuli, just as the experience of concreteness is projected to promote expectations of nearness. And by a reciprocal process again, the experience of psychological distance leads to expectations of high-construal level, just as the experiences of nearness cue low-construal expectations. In the words of two leading CLT theorists, ‘different levels of construal serve to expand and contract one’s mental horizons and thus mentally traverse psychological distances’.10

Some examples should make these claims clearer. Take the high-construal noun ‘civilisation’: typically, this prompts one to expect psychological distance along several dimensions—thus, one speaks of ‘Roman civilisation’ (temporal distance), ‘Japanese civilisation’ (spatial distance) or ‘alien civilisation’ (social distance), but never ‘Oxford civilisation’. In the other direction, distance cues high-construal expectations. Probabilistically remote events, for instance, prompt low-detail hypotheticals (‘if alien civilisations exist, we will discover whether there are cultural universals’), when likely events enjoin more detailed ones (‘if there’s traffic on Oxford High Street, I’ll divert via Queen’s Lane’). Similar considerations obtain with respect to low-construal stimuli, though here of course the relation is with nearness. For example, a detailed statement like ‘The venue for Julia’s vocal performance has poor acoustics’ is typically consonant with Julia’s recital happening soon and not in three decades, just as ‘Julia hopes to be still able to sing on her 100^th^ birthday’ does not usually enjoin one to ask whether the room in which she sings will have air conditioning.

In themselves, these examples are merely suggestive; the achievement of CLT is to support them with an experimental programme that links a diverse range of behavioural and cognitive outcomes to the experience of both psychological distance and construal level. With respect to distance, the evidence is that framing actions as far away on one of the dimensions of psychological distance makes high-construal purposiveness salient while inhibiting considerations of low-construal context. Liberman and Trope,11 for instance, show that action statements like ‘locking a door’ are represented in terms of constituent subactions (‘putting a key in the lock’) when located in the imminent future, but in terms of agentive intention (‘securing the house’) when set a year from the present. Cognate results are available for spatial distance,12 probabilistic distance13 and social distance.14 Another effect of distance is to prime for morality over empathy (ie, the application of abstract normative principles over the emotional engagement with the needs of another). In this connection, Eyal, Liberman and Trope demonstrate that the violation of widely accepted moral rules (sibling incest, eating pets, desecrating national symbols) is judged more harshly (ie, more morally) in far-future conditions than in near-future ones15 —presumably because contextual mitigating factors are not in play. Intensity of emotion is also linked to distance cues, with events like visiting the dentist being imagined to be less intense when imagined to be further in the future than near to the present;16 similarly, objects and events moving away from the subject are felt as having less emotional valence than object and events moving towards.17 Some other behaviours that have been identified as affected by considerations of construal level and distance include the propensity to conform,18 the alignment of goals with desires,19 the perception of expertise,20 the experience of shame,21 the appreciation of creativity,22 the use of politeness23 and the making of economic decisions.24 In all cases, the general result is the same: abstraction and psychological distance are cues for one another, just as concreteness and psychological nearness are also.

As indicated, the link between these considerations and the mood disorders comes by way of attentional capture in depression and anxiety. Though both conditions share a common cognitive trait in overrumination and are thus often comorbid,25 the focus of rumination tends to be quite distinct—and this is where CLT considerations come into view. Starting with depression, this should be understood here to mean ‘major depressive disorder’ (MDD) or ‘persistent depressive disorder’ (PDD) as classified by the *Diagnostic and Statistical Manual of Mental Disorders, Fifth Edition* (DSM-V). Typical symptoms of both involve depressed mood, diminished interest in activities, weight loss/gain, insomnia/hypersomnia, psychomotor agitation/retardation, fatigue, feelings of worthlessness and/or guilt, lack of concentration or decisiveness, and recurrent thoughts of death.26 MDD requires more symptoms for a shorter period for diagnosis; PDD requires fewer symptoms for longer. Both are to be distinguished from bipolar disorder, which the *DSM-V* now classes alongside the psychoses.27 With respect to the phenomenology of both conditions it would seem that the ruminative emphasis falls on capture by high-construal, negatively valent thoughts and memories. For instance, Watkins and Teasdale note that ‘overgeneral memory is a disorder specific phenomenon found in depression and posttraumatic stress disorder but not in generalised anxiety disorder, social phobia or obsessive-compulsive disorder’,28 with Watkins amplifying this into the more general claim that ‘the level of goal/action identification adopted in major depression is abnormal and dysfunctional, with patients with depression tending to adopt more abstract levels of goal/action identification, at least for negative information, than non-depressed controls’.29 Significantly, the CLT-predicted distance priming that this experience of abstraction should enjoin can be readily identified in first-hand accounts of depression. Matthew Ratcliffe, for example, collates the following descriptions: ‘There was just an unfathomable distance between me and any other human being’;30 ‘It feels as though you’re watching life from a long distance’;31 ‘the world looks different—familiar things seem strange, distant’;32 ‘things are no longer appear available; they are strangely distant’;33 ‘I was terribly alone, lost in a far-away place’;34 ‘You’ve lost a habitable earth. You’ve lost the invitation to live that the universe extends to us at every moment’.35 There can be no doubting the salience of psychological distance in these reports.

Moving on to anxiety, this condition should be taken here as a proxy for ‘generalised anxiety disorder’, which the *DSM-V* defines as ‘excessive, uncontrollable anxiety or worry for at least 6 months and associated with two of restlessness, fatigue, irritability, muscle tension, and sleep disturbance’.36 At first glance, the literature seems to identify these symptoms as a form of attentional capture by high-construal stimuli. Watkins, for instance, concludes that generalised anxiety disorder (GAD) is ‘characterised by a predisposition towards adopting an abstract level of goal/action identification as the prepotent operational level … patients with GAD demonstrate a biassed tendency towards a more abstract level of processing’.37 However, though this formulation may be technically correct—after all, it is certainly true that worry deals in hypotheticals—it is questionable how useful it is. (And note the disagreement with Watkins and Teasdale quoted above.)38 Instead, it would seem more accurate to characterise anxiety as the making concrete of ostensibly abstract threats. Mathews, for example, notes that anxiety is marked by ‘persistent awareness of possible future danger, which is repeatedly rehearsed without ever being resolved’39—an appreciation that is echoed in a number of studies that identify anxiety as a form of ‘selective processing that fits [the anxious individual’s] view of the world as dangerous’.40 From a CLT perspective, this amounts to collapsing the psychological distance on the probabilistic dimension. By making unlikely events—that is, events that are probabilistically distant—salient, anxiety cues the collapsing of mental horizons into the here and now.

A cursory examination of first-hand descriptions of anxiety bears out this view of anxiety as an overconcrete immersion in the near-hypothetical. Though equivalent collations of anxiety experiences to those available for depression are less easy to find, discussions on social media forums offer some useful evocations. For instance, a psychiatrist on Reddit recalls a patient as saying anxiety ‘feel[s] like she tripped and the moment where you don’t know if you are going to catch yourself or not is how she felt all day long.’41 Another commenter likens it to what happens when your ‘stomach turns and you get hot and your heart pounds, whatever happens when you think something went very wrong and you’re about to catch shit for it’;42 a second exhorts the reader to ‘Imagine if every small decision had life or death consequences’.43 An author on the *Anxiety and Depression Association of America* website recalls, ‘I’d be stuck in traffic and these irritating voices would take my brain hostage. “Did you leave the coffee on? The house will catch fire, the neighbours will burn!”’44 Another recounts, ‘I don’t see five to ten ants. I see the inevitable 100 to 200 that I imagine will invade and eventually carry off our house. It’s very hard for me to deal with the here and now when I am catastrophizing’.45 The examples could be multiplied, but the point is clear: anxiety, contra depression, shrinks the horizon of cognition into immediately nearby spatial and temporal concerns.

The question that follows from framing mood disorders in this way is whether doing so provides any therapeutic insights into anxiety and depression. The answer would seem to be that it does, even if such insights are not explicitly articulated using CLT.46 Specifically, there is a small (but growing) body of evidence that exposing the depressed and the anxious to stimuli that exhibit a construal level opposite to that which characterises their condition yields positive therapeutic results. In the case of depression, Werner-Seidler and Moulds47 establish that prompting depressed and recovered individuals to recall an autobiographical memory in abstract and concrete modes yields a positive impact in the concrete mode; in their words, ‘a concrete processing mode enabled positive memory recall to have a reparative effect on mood for depressed and recovered participants’.48 Equivalently, Watkins *et al* show that complementing treatment as usual (TAU) for depression with concreteness training ‘seems to be an efficacious treatment for mild to moderate depression in primary care, producing significantly better outcomes than TAU’.49 Viewed through the lens of CLT, these results are explained by the adopting of a low-construal cognitive frame, where the depressive tendency towards ruminative overgeneralisation is recalibrated towards shorter psychological distances. These distances, being more amenable to everyday projects and goals, challenge the impotence in the face of the cosmos that characterises depression.

With respect to anxiety, the situation once again seems less clear-cut. Amir, Beard, Cobb and Bomyea demonstrate that diverting attention away from threatening stimuli succeeds in reducing anxious feelings50 —a result that is replicated in several places across the literature.51 However, there is also evidence that actively exposing anxious individuals to inducing stimuli can, over the course of time, ameliorate symptoms.52 How can these different data be reconciled? The key to doing this comes with recognising the role played by abstraction in both protocols. Attention diversion techniques invariably work by training participants to attend to neutral stimuli over threatening ones; as such, they strip away one dimension of experience—valence—and thereby shift the individual’s perception of the world into a marginally more abstract mode. It can be argued that exposure therapies do the same, but in a radically different way: by inserting the threatening stimulus into a series of encounters, they dampen its singularity and subordinate it to an abstract category that is amenable to cognitive manipulation. What emerges, therefore, is the finding that ‘a tendency to preferentially attend toward or away from threat, relative to an equal distribution of attention irrespective of threat, results in greater reduction in clinical symptoms and diagnoses’.53 In other words, so long as a high-construal perspective is adopted—whether by diversion or habituation—the anxiety-inducing character of a stimulus will tend to diminish.

In sum, there exist reasonable grounds for thinking that the results of CLT have relevance for the treatment of both depression and anxiety. I stress, once more, that this does not amount to saying that they are explained by CLT, or that CLT captures the full phenomenology of either condition. If it did, it would be a damning verdict on psychological medicine for it to have missed so straightforward a result, given the amount of research that anxiety and depression have stimulated over the years. Instead, the value of the CLT perspective is that it allows for connections to be made with cultural processes that may have a therapeutic value for both conditions. These processes will be the target of the next section.

## Construal level and culture

Viewing culture through the lens of abstraction is not a new idea. As long ago as 1908, Wilhelm Worringer wrote of ‘the antithetic relation of empathy and abstraction’, and how the impulse towards abstraction ‘finds in beauty in the life-denying inorganic, in the crystalline or, in general terms, in all general law and necessity’.54 For Worringer, the motivation towards abstraction is ‘the immense spiritual dread of space’,55 which leaves humanity ‘tormented by the entangled inter-relationship and flux of the phenomena of the outer world’.56 By positing abstraction as the antidote to the anxiety entrained by the unpredictability of the world, it should be clear that Worringer is making a connection between mood and forms of cultural representation that is cognate to the issues under discussion here.

In the present section, my aim is to go beyond Worringer’s purely qualitative appreciation of how abstraction shapes the experience of culture and develop quantitative metrics that allow for such abstraction to be measured. The value of such measurement is that it allows for cultural representations to be compared with one another, both within and between the categories they belong to. This is a potentially controversial move, given the long-standing resistance to such acts of quantification in the humanities and the interpretive social sciences.57 Nevertheless, it is to be hoped that recent work in areas like cognitive cultural studies will have gone some distance towards illustrating the power of using empirically informed methodologies to engage with the symbolic apparatus of culture.58 Equally, the movement towards the experimental humanities points to the value of having manipulable measures that allow for the testing of predictions against real-world audiences.59 Here, both considerations will allow for an account of different forms of cultural representation that predict their impact on the mood disorders.

At a practical level, I shall focus mostly on construal level as my variable of interest. This is not at all to dismiss psychological distance as playing an important role in the operations of culture; indeed, I have written previously on how manipulations of distance play a crucial role in establishing the cognitive effect of genres such as science fiction.60 Instead, my view is that the necessarily synoptic exercise of surveying how distance emerges in several media would eclipse the no less important (and comparatively underdeveloped) issue of abstraction, which must therefore be my principal focus here. Saying this, however, invites the question of how construal level can be quantified. While it is often intuitively obvious whether a representation is abstract or concrete, this is of little value when precise comparisons are needed. Moreover, the experimental manipulations of construal level in the literature, though fit for purpose, are not generalisable across multiple modes of stimulus presentation. What is therefore needed is a reliable proxy for construal level that yields precise values and can be extended across a variety of media.

I propose here to use Shannon entropy as this measure.61 Like the thermodynamic concept it is derived from, Shannon entropy (hereafter just entropy) is a measure of how much structure is required to encode a message, with high entropy messages being more unpredictable than low entropy messages. As the central concept in information theory, entropy is surrounded by a forbidding formal vocabulary. Nevertheless, it is relatively easy to illustrate by way of a linguistic example. For instance, it is easy to see that asking someone to complete a three-word phrase that starts with ‘the northern’ will more often produce ‘the northern lights’ or ‘the northern hemisphere’ than ‘the northern engine’ or ‘the northern babysitter’. This is because, in English, the former two phrases occur with a much higher frequency than the latter, meaning that any guess concerning how the phrase should be completed is more likely to be correct if it uses ‘lights’ or ‘hemisphere’ as the relevant word. In this sense, the phrase ‘the northern lights’ has less entropy than ‘the northern babysitter’, as it would require fewer guesses to reconstruct it in the event of the last word being missing.

In more formal terms, entropy is therefore ‘a measure of the information contained in a message as opposed to the part of the message that is strictly determined (hence predictable) by inherent structures’.62 What this involves in practice is calculating the average freedom to vary each symbol or item in a given message relative to the constraints that operate on that message. This is done using the following formula, where *H* corresponds to the entropy of the message *X*, *i* denotes a value in the range 0≤ *i* ≤ k when *k* is the number of symbols in the message, and *p*(*x_i_*) is the probability of a specific symbol *x_i_* occurring:

$$H(X)=-\sum_{i}p(x_{i})log_{2}p(x_{i})$$

For those unfamiliar with the formalism, the sigma operator ‘Σ’ means that if there are *k* symbols, then the entropy should be calculated for each one of them and the results added together. The log_2_ *p*(*x_i_*) quantity picks out a number *y*, such that 2*^y^*=*p*(*x_i_*). Though this formula outputs a value in bits for any well-defined system, this number is often normalised so it can only take values between ‘0’ and ‘1’ by dividing by the quantity log_2_(*k*); this quantity is called metric entropy. To see how the formula works in advance of its use below, take a simple example: a coin toss with an unbiased coin. There are two possible results (heads or tails) with the same probability each; thus *p*(*x_i_*)=0.5 for *i*=1 and *i*=2. The entropy of the system is calculated by summing across both possible results: – [(0.5 × log_2_(0.5) + (0.5 × log_2_(0.5)]=1. Thus, this system can be completely described using 1 bit of information. (Note that the metric entropy is also one in this example, as log_2_ 2 =1.)

The relevance of entropy for the discussion of construal level and culture is that it reliably tracks abstraction. The core cognitive operation of abstraction is to strip away redundant detail and make a given system predictable—a function that reaches its apotheosis in mathematics and logic, but which is present in any act of classification or generalisation. Conversely, concreteness entails unpredictability, with its limit case being a purely random distribution of elements where the only structure is that described by the normal distribution. In the terminology under discussion, this means, counterintuitively, that *low* entropy corresponds to *high-*construal level and *high* entropy corresponds to *low-*construal level. If this identity is allowed, it means that entropy can be used as a proxy for identifying the likely impact of cultural representations on anxiety and depression.

Naturally, all of this raises the question of what such analysis might look like in practice. I shall initiate it now by giving an overview of how three modes of representation—image, narrative and film—can be discussed in terms of construal level. I would like it to be noted from the outset that my treatments of each area will be highly summary in character, and for every operational choice I make, I acknowledge that a specialist will be able to make the case for a different one. In defence of my procedure, I state here that my aim is only to demonstrate *in principle* how one might approach these three areas. Any thoroughgoing practical demonstration would need to be more circumspect in its approach, if also longer than is useful for an article-length exposition.

### Image

Most discussions of the therapeutic potential of image focus on the production rather than the reception of visual artworks.63 However, as Vidler notes ‘anxiety, estrangement, and their psychological counterparts, anxiety neuroses and phobias have been intimately linked to the aesthetics of space’.64 For Vidler, this is a specifically modern phenomenon, but it can readily be seen that the attempt to ‘permeate the formal with the psychological’65 can inhabit the visual art of any period. The 70 k BCE ochre carvings from the Blombos Cave, for example, display a geometrical linear styling that has no obvious mimetic intention;66 conversely, the 17 k BCE animal paintings of the Lascaux Cave evince a highly concrete realism.67 Closer to the present, Celtic art of the Hallstatt style (1200–500 BCE) is geometrical, linear and spare, while that in the La Tène (450–1 BCE) style is curvilinear, richly detailed and organic68—no less than the formal innovations of the twentieth-century avant-garde contrasts with the nineteenth-century pictorial realism. The point is that the oscillation between mimetic detail and formal abstraction is a pattern the repeats across the history of visual culture.

**Figure 1:**
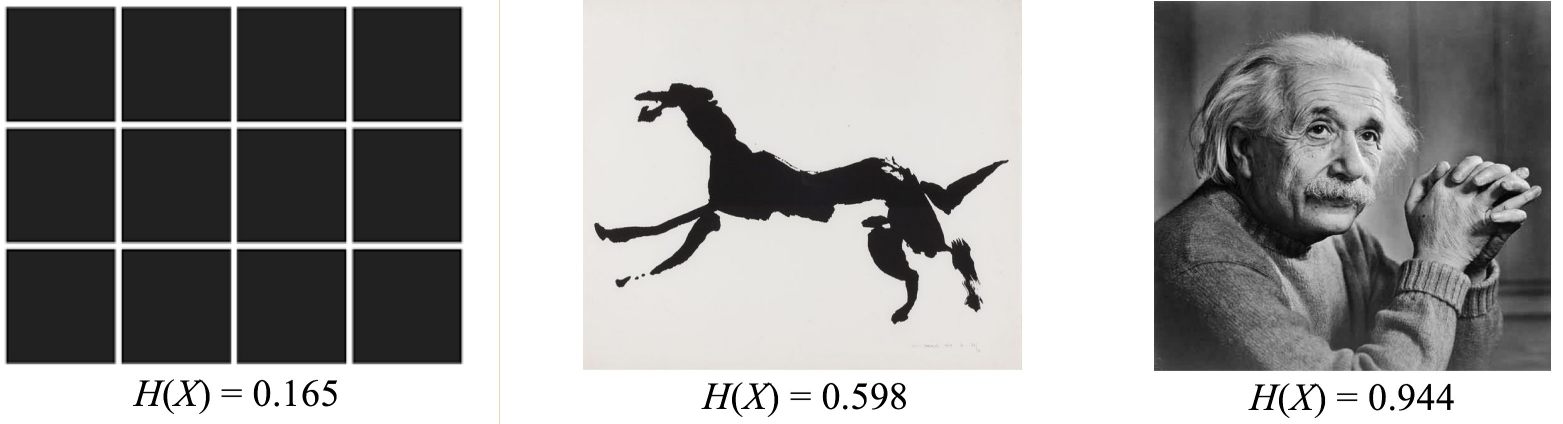
Metric entropy scores for three images, where ‘0’ is the lowest possible value and ‘1’ is the highest. The rectangular grid is highly predictable and has a low score. The middle image—taken from Louis le Brocquy’s illustrations to Thomas Kinsella’s translation of the medieval Irish epic, Táin Bó Cuailgne—blends pictorial and schematic elements, and takes a value in the middle of the distribution. The final image, as a photograph, lacks any easily defined organising principle, and thus comes close to the entropy of a purely random distribution of pixels (ie, a score of 1).

To the extent that this is so, entropy should provide a reliable measure of abstraction in an image. The question concerns how it might be calculated. The standard protocol for doing this is to use colour as the variable *i* in the entropy formula, such that it takes a value from the set of *k* colours that make up an image. The object taking the value can be thought of as the pixels making up the image, so that for any colour *i*, a given image will contain *m* pixels of that colour. Thus, for any randomly chosen pixel from the total of *n* pixels in the image, the probability of it being colour *i* will be the fraction *n*/*m*. To illustrate, take an 8-bit grayscale computer image: this format allows 256 (ie, 2^8^) shades of grey that can occur in any admixture. The probability of a randomly selected pixel exhibiting any given one of these shades will be given by dividing the total number of pixels exhibiting the shade by the total pixel count of the image. In turn, this allows the image entropy to be calculated by summing the 256 products of each shade’s probability by the log base 2 of this probability—a number that can then be normalised by dividing by log_2_(256). As even small images have very large numbers of pixels, it would be impractical to reproduce such a calculation here; it is easier just to show the results generated for specific images (figure 1), which I calculate using a python function specifically scripted for the task. As can be seen, there is a clear linear progression such that the more abstract an image is, the lower the entropy score.

Directing these results back to CLT, what can be said is that entropy tracks abstraction—and therefore construal level—in images in a reliable way. High-construal images, in virtue of exhibiting law-like regularities, are predictable and have low entropy; conversely, low-construal images frustrate schematic perception and have high entropy. The prediction that follows is that high entropy images should be of value for individuals with depression, when low entropy images should be of value for those suffering from anxiety. Such a claim invites assessment: is it the case that entropy tracks the therapeutic value of images?

To the extent that there is evidence available, it would seem that it does, looked at from both a historical and an experimental perspective. Starting with anxiety, Currey and Kasser69 compare the therapeutic impact of colouring a mandala design, colouring a generically abstract plaid design and colouring by way of a free-form unguided exercise. The results clearly show that colouring low entropy images in the form of the plaid design and the mandala reduced anxiety in a statistically significant way relative to colouring the free-form design, which has no impact—with this outcome being replicated in an independent follow-up study.70 In a separate study, Sandmire, Gorham, Rankin *et al* 71 compare the impact on anxiety of mandala design, free-form painting, clay sculpting, collage making and drawing. Small sample sizes by condition did not allow the different interventions to be statistically distinguished. Nevertheless, given that the largest group chose the mandala design, the positive results they establish for art therapy across all conditions are consistent with the CLT abstraction hypothesis. Though more work is needed, these results clearly point to high entropy images as having a potentially positive impact on anxiety.

With respect to depression, the evidence is that the types of images produced by depressed individuals are of a low entropy kind, to the extent that they use less colour, have more empty space, are more constricted, are less effortful and are less meaningful.72 Correspondingly, it is unsurprising that high entropy images appear to be more therapeutically efficacious for depression. For instance, in a study that involved patients with cancer in a non-abstract watercolour-based art therapy intervention, Bar-Sela, Atid, Danos, Gebay *et al* 73 found that depression was reduced while anxiety levels remained the same. Beyond this, it is difficult to generalise, as most experimental studies do not distinguish between the specific types of art therapy administered. Nevertheless, it is possible that the relative preponderance of concrete and mimetic modes of representation in art therapy is responsible for the positive impact on depression identified in several meta-analyses.74

It would seem, then, that there is evidence in support of the view that CLT effects on depression and anxiety can be tracked using entropy as a proxy, and they take the direction predicted. Necessarily, this requires further testing if the hypothesis is to receive the support it requires. Equally, a more cognitively ‘thick’ account of visual perception is needed to allow for entropy-reduction processes (like face and scene recognition) that improve the predictability of images. Nevertheless, it should be evident that the CLT-entropy approach points to a potentially important line of inquiry for the discussion of the therapeutic impact of images.

### Narrative

If art therapy has an established therapeutic pedigree, treatment using literary materials has emerged in the last number of years as a parallel method of engaging with various disorders.75 Variously termed ‘book therapy’ or ‘creative bibliotherapy’, the idea is that ‘active immersion in great literature can help relieve, restore, or reinvigorate the troubled mind’.76 Though certainly an appealing prospect, the field is new and there is no robust proposal as to why literature should have a therapeutic effect in the first place. Montgomery and Maunders probably offer the most considered approach when they argue for literature as a vehicle for Cognitive Behaviour Therapy (CBT) effects,77 though this ultimately relies on the same ideas concerning readerly identification in fiction that underwrite less developed approaches.78 Moreover, even allowing that literature has a therapeutic effect, no approach predicts how different types of literature will interact with any one of the 297 or so conditions that at least one diagnostic system identifies as defining the repertoire of mental illness.79

As scoping a response to these problems for all forms of literature is obviously impractical, I shall restrict myself here to outlining how a CLT-informed entropic perspective might be used to make predictions concerning the therapeutic impact of narrative literature. Naturally, this poses the problem of how narratives can be understood as ‘abstract’ or ‘concrete’—not least because there are several levels, ranging from the lexical to the sentential to the discursive, that may exhibit such qualities. One useful concept for probing the distinction is that of *narrativity*.80 This is a global term that indexes the extent to which a cultural artefact lends itself to narrative elaboration across all its elements. A text with a high degree of narrativity will exhibit a judicious mix of surprise and predictability, with a preponderance of either surprise or predictability being deleterious to a text’s narrativity. As David Herman notes, ‘there is a lower limit of narrativity, past which certain ‘stories’ activate so few world models that they can no longer be processed as stories at all’, just as there is ‘an upper limit of narrativity, past which the tellable gives way to stereotypical, and the point of a narrative, the reason for its being told, gets lost or at least obscured’.81 In other words, a highly concrete text will be one that frustrates narrativity by resisting predictability, while an abstract text frustrates it by being too general. The connection here with entropy is obvious: texts that are either too high or too low in entropy are poor in narrativity.

One way in which these distinctions can be illustrated is by way of the link between character and action—a central plank of all forms of narrative. In this regard, one achievement of formalist models of narrative has been the identification of narrative ‘grammars’, where the key intervention involves abstracting recurrent classes of action from a given class of narratives to arrive at a general model. These models often generalise across categories like region and genre,82 but there also exist accounts that identify features purportedly present in all narratives.83 Their value for the present purpose is that they offer one clear dimension on which narrativity varies, to the extent that texts that entirely coincide with, or entirely deviate from, narrative grammars are also poor in narrativity. I shall illustrate this here using A J Greimas’s actantial model84 —not out of any conviction that Greimas is correct where others are wrong, but simply because his model is well known and supports a formal demonstration that can be readily extended to other models.

**Figure 2:**
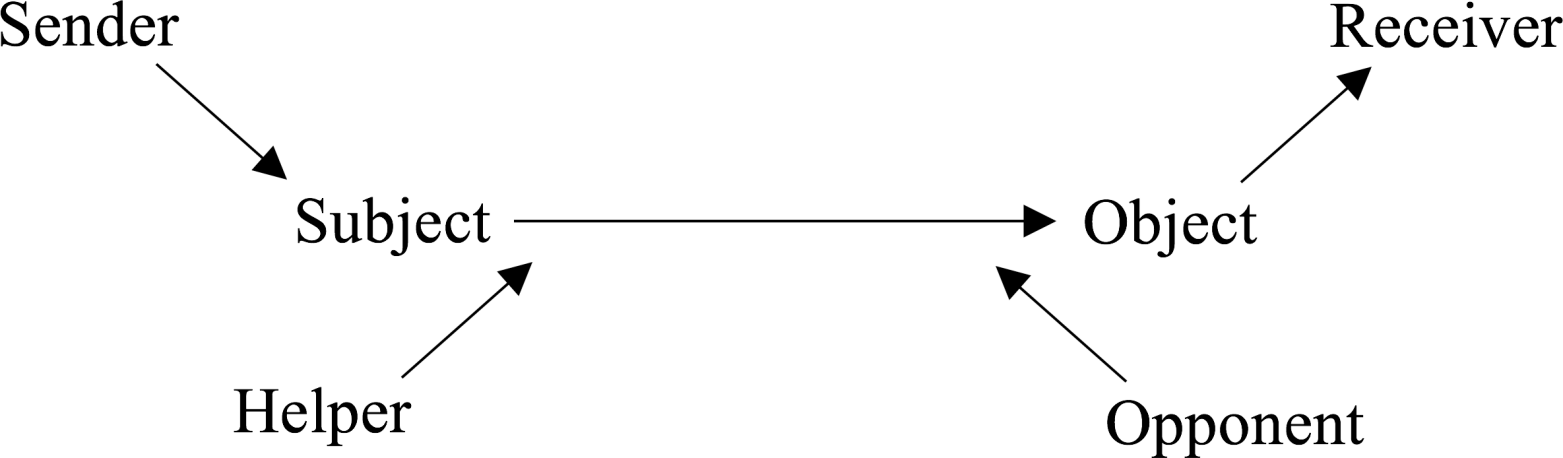
Greimas’s six-term actantial account of narrative.

For Greimas, a character in a narrative can take one of six actants or roles: it can be a *subject*, an *object*, a *helper*, an *opponent*, a *sender* or a *receiver* (figure 2). The subject and object are paired in a relation of desire, want or need, with the subject exercising this relation on the object. Take Ian Fleming’s *James Bond* novels: here, Bond is typically the subject, with the defence of democratic values and the social order being the object. The helper and opponent are paired in a relation of power, such that they either augment or detract from the subject’s ability to pursue the object. In the Bond narratives, the helpers can be Q, the Bond girl, Felix Leiter and so forth; the opponent is manifested through the agents of the USSR and the various criminal enterprises that Bond confronts. The sender and receiver are paired in an axis of transmission, where the sender legitimates the subject’s actions, the value of which is delivered to the receiver. Thus, M, as representative of the Crown, authorises Bond’s actions, which benefit the British public and the wider democratic world. According to Greimas, these categories are present in all narratives, such that any character can be identified with a given actant on a given appearance (ie, they can be identified with different actants at different points in the narrative). Thus, what the actantial model defines is an underlying principle of organisation that maps the information contained in the set of character appearances into the information contained in the set of actantial roles.

Viewing this model through the lens of entropy, the six actants define a set of values that partition the set of character appearances. This means that, for any given character appearance, that appearance can be classified under a category *i*, where *i* belongs to the set *(sender*, *receiver*, *subject*, *object*, *helper*, *opponent)*. The sum of the character appearances in each category will then total the amount of appearances in the narrative. The probability, *p_i_*, of a given appearance being a particular actant is found by dividing the number of appearances in that category by the total number of appearances. As per the formula, the entropy is calculated by summing the products of each probability *p_i_* by log_2_(*p_i_*). In the case where there is an equal likelihood of a character belonging to one of the six roles in a given appearance, the metric entropy of the narrative is exactly 1, as there is no structure present. The latter enters into the picture via genre, which will foreground and background different actantial roles, thereby changing the relative probabilities involved. In Greimas’s words, ‘an articulation of actors constitutes a particular *tale*; a structure of actants constitutes a *genre’*.85 To properly represent this would require exact data on the relative proportion of actantial roles in different genres, which it is beyond the scope of the present article to provide. Instead, it is more useful to look at how entropy and narrativity relate to these considerations of abstraction and concreteness.

In this regard, the tendency towards concreteness has a clear expression in the realist novel. As an exercise in ‘the mimesis of consciousness’,86 realism participates in the unpredictability of its subject matter. That is, by attenuating the connection between actantial role and character identity in favour of character identity, narrative realism refuses the predictability of a schema and makes salient instead the sense of subjective freedom that attends personhood. Given the social orientation of much human cognition,87 it is unsurprising that that texts exhibiting this tendency should be narrative to a high degree. Nevertheless, it is no less true that the wholesale reproduction of subjective experience as found in texts like James Joyce’s *Finnegans Wake* 88 or Virginia Woolf’s *The Waves* 89 have the effect of reducing narrativity. By requiring the reader to invest sustained cognitive labour before she can extract the general principle from the concrete particular, they frustrate the sense of (immediate) universality that is implicit in highly narrative texts.

On the abstract side of the spectrum, quasi-formulaic narratives like folk tales and myths are close mirror images of realist fiction, in that they also have psychological verisimilitude and functional roles, even if here the functional role predominates. The result is that such genres evince the same degree of narrativity as realist fiction, while having lower entropy. The ‘pathological’ tail to this end of the narrativity distribution is probably to be found in those simplified narratives that entirely dispense with specific actants. These are most commonly found in children’s discourse, which evolve over the course of development from primitive assertions of desire or causality to fully worked-out stories.90 Notably, such narratives are also visible in the ‘anti-narratives’ of modernist fiction, which strategically interrupt the story-telling process so as to highlight its inadequacy to human experience. Take, for instance, Samuel Beckett’s trilogy of novels, *Molloy*, *Moran* and *The Unnamable*, where all distinctions of character and structure eventually collapse into the ‘Where now? Who now? When now?’ of *The Unnamable*’s immobile subject.91

The general picture that emerges from this is that there is a clear alignment between the concept of entropy under discussion here and narrative structure. Moreover, this alignment is not merely theorised to exist, but has a long-standing expression in narratological terminology. But while this is welcome and satisfying, it is of incidental importance to the present inquiry if it cannot be connected with anxiety and depression. In this connection, the evidence is promising, if limited. As might be expected, the difficulty of making lengthy narrative materials amenable to experimental manipulation means that such studies as exist tend to focus on shorter narratives or narrative selections. With respect to depression, Carney and Robertson show that individuals exhibiting traits that correlate with depression are more strongly engaged by a high entropy version of a narrative text than a low one.92 On the side of anxiety, Glavin and Montgomery93 and Montgomery and Maunders94 offer a meta-analyses of studies that use literature in the treatment of various conditions and find either modest effects or no effects, but suggest that lack of comparability and poor design make definitive conclusions untrustworthy. Orthogonal to both, Troscianko shows substantial effects for fiction reading on several dimensions of behaviour relating to eating disorders, but the classification of fiction type is determined by thematic focus rather than abstraction or concreteness.95 Beyond this, the research literature offers little in the way of robust empirical evidence, even if there is no shortage of speculative pieces.

Thus, though there is no evidence against the proposal that abstraction impacts differentially on mood disorders, and some small evidence for it, a programme of testing is needed to properly establish the effect of narrative abstraction on depression and anxiety. I am currently engaged in an ongoing programme of such testing, which aims to ground the efflorescence of interest in creative bibliotherapy with solid empirical data. As yet, however, no solid conclusions can be reached concerning the role of abstraction in the cognitive impact of fiction—even if there are reasons to be hopeful that it might yet be used to good therapeutic effect.

### Film

Just as literature has been suggested as a possible vehicle for therapeutic intervention, so too has film. The practice of using ‘motion pictures in a structured manner with specific populations for particular therapeutic gains’96 dates back to at least the 1940s,97 and continues today in a wide range of healthcare contexts.98 To the extent that it is theorised, ‘cinematherapy’ is framed in much the same way as its literary equivalent, with many theorists even going so far as to explicitly identify it as a form of bibliotherapy.99 Given the problems with bibliotherapy, this does little to clarify how cinematherapy might work, but it does point to CBT-style effects as the most commonly assigned mechanism.100 Though this may well be the case, the fact remains that such accounts say little about the specifically filmic dimension of cinematherapy, or indeed how different types of film relate to different conditions.

As will be anticipated, my suggestion is that abstraction provides one way of approaching this issue. In fact, given that film contains both visual and narrative components, it can be readily seen that two ways in which filmic entropy can be measured have already been covered. The task comes with outlining how these approaches might be adapted to account for the specific effects of cinema. In the former scenario, this involves extending the static entropy measure of a still image into a dynamic register of multiples of images per second; in the latter, it means incorporating the issues that arise when characters and actants are instantiated in physically perceivable agents and objects (ie, actors and props). Once both issues are dealt with, it should then become possible to consider the therapeutic impact of film.

**Figure 3:**
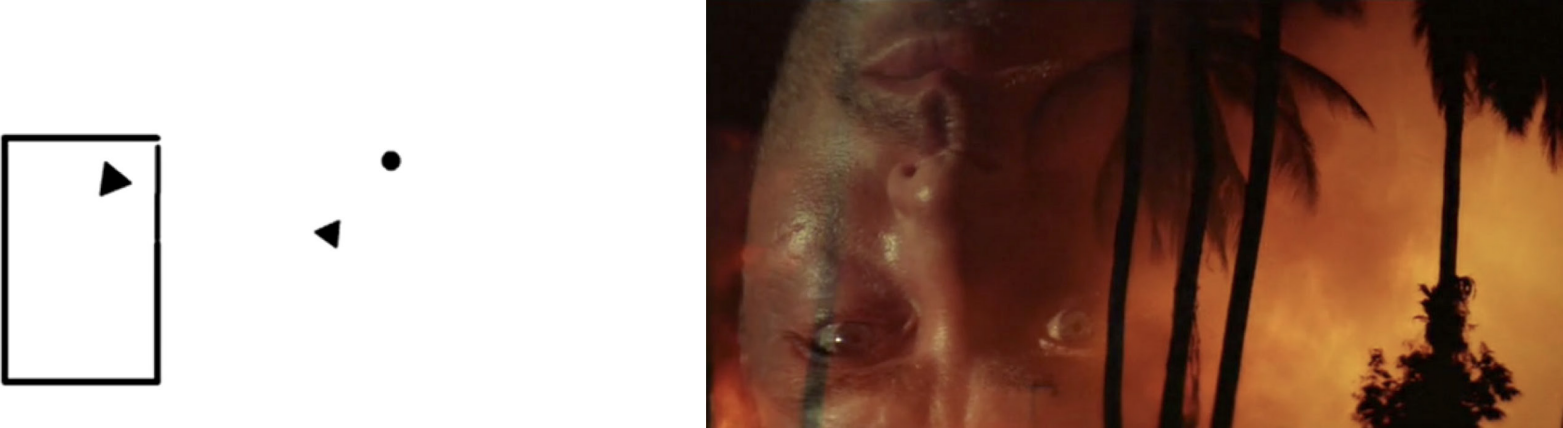
Illustration frames from the Heider-Simmel demonstration frame (left) and *Apocalypse Now*.

**Figure 4:**
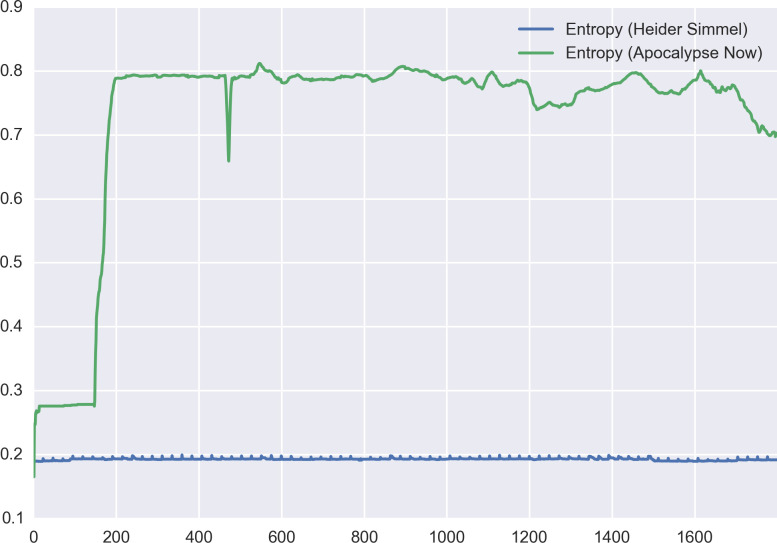
Entropy by frame for Heider-Simmel and *Apocalypse Now*.

**Figure 5:**
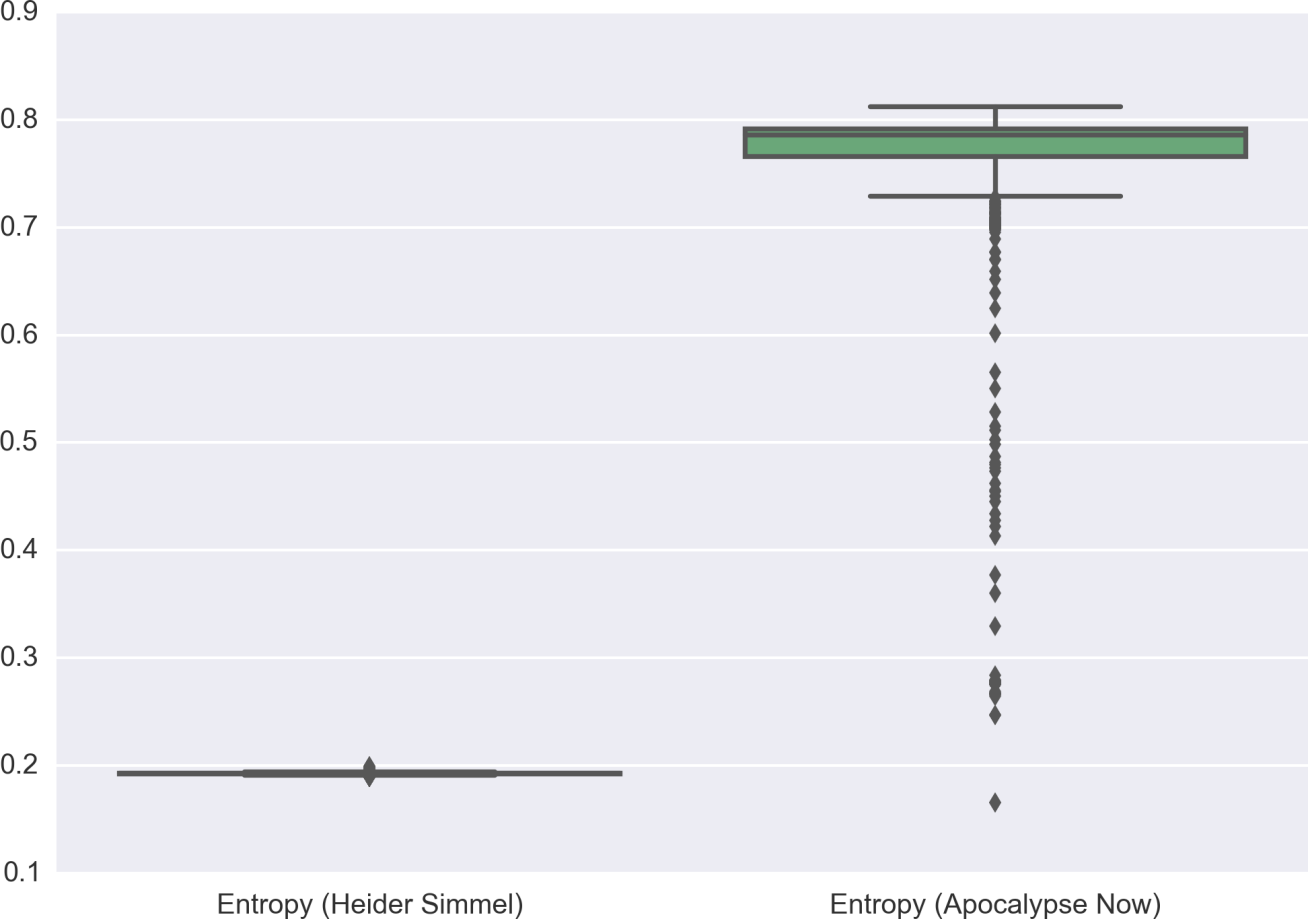
Box plots for Heider-Simmel and *Apocalypse Now*.

To start with the issue of dynamic entropy, there is an obvious sense in which the relevant abstraction measure for film is not the average entropy across its frames, but the distribution of the entropy values these frames take. Take, for instance, the Heider-Simmel demonstration,101 which is the famous animation that uses moving shapes to illustrate the human propensity to assign agency to objects. Consider, at the same time, the introductory sequence to Francis Ford Coppola’s *Apocalypse Now*,102 which uses richly worked cinematography to conflate the external chaos of the Vietnam war with the inner turmoil of its protagonist, Captain Willard (figure 3). It should be clear that, on a frame-by-frame basis, both films will exhibit very different entropy measures—as is clear from plots of the entropy levels for the first 1800 frames of each (figure 4). Equally, however, what should emerge is that there are marked differences in the *variance* of the entropy across both films. Though neither exhibits a particularly large variation, the overall spread of entropy in *Apocalypse Now* is much wider than in Heider-Simmel, and would likely be wider again in a longer selection (figure 5). What this serves to show is that a measure like the IQR (the numerical interval in which the middle 50% of the data lie) is likely to offer the best measure of the entropy of a sequence of film. Where this range is narrow and lies close to 1, the film will be concrete and exhibit high entropy; where it is narrow and closer to 0, it will be abstract and have low entropy. Where there is a large IQR, there is likely no worthwhile measure of the central tendency of entropy, and analysis will have better results by focusing on shorter subsequences.

Moving on to the issue of character-based entropy, the specifically filmic problem that emerges is the high cognitive load that the audience must sustain when they are inferring the motives of ostensibly unknown persons. Unlike a linguistically mediated narrative, where mental states can, in principle, be directly communicated, acted roles are physically instantiated in real people and thus engage computationally expensive inferential processes.103 As pushing cognitive load beyond a certain point is typically antihedonic,104 this means that film, as a genre, perpetually runs the risk of alienating its audience due to excessive entropy. How might this be resolved? One solution comes by way of the ‘star’ phenomenon. As noted by theorists such as Richard Dyer, ‘The star’s presence in a film is a promise of a certain kind of thing that you would see if you went to see the film’.105 Though Dyer volunteers this as motivated by economics, it functions equally well to reduce entropy. That is, if the presence of a specific actor entrains a set of performance expectations, then these expectations reduce cognitive load by creating a set of priors that prestructure the relevant inferences. Putting this in terms of the entropy formula, the variable *i* will thus pick out a member of the set of conventional movie roles *(action hero*, *femme fatale*, *comic relief*, *maverick*, *wise counsellor*, etc). Summing across his or her appearances, the presence of a specific actor will increase the conditional probability of a given character belonging to a given role, thereby providing implicit context for making sense of their actions. As this serves to increase predictability, it lowers overall entropy, and helps resolve the problem of cognitive load. In this sense, the star system created by the film industry operates as an entropy reduction ‘grammar’ that increases consumption by lowering the cognitive demands of understanding the actions of person-like agents.

Bringing these considerations back to the mood disorders, what emerges is that abstraction is concomitant with films that have predictable action structures and character motivations, and which impose a low perceptual processing burden. Conversely, detailed visual innovation and eschewal of ready-made associations between actors and roles creates a highly concrete cinema that resists predictability. Once more, the question is whether the latter type of cinema is therapeutically efficacious for depression, where the latter is for anxiety. Such experimental testing as has been conducted supports the general idea that cinema can have a therapeutic impact, though there are no data that can be parsed in terms of abstraction and concreteness in cinematic representation and how they serve to differentially impact on mood disorders. Thus, even more than image and narrative, cinema remains a *terra incognita* with respect to its clinical impact. It remains possible—even plausible—that it has a palliative effect on conditions like depression and anxiety, but this needs to be supported by well-designed experiments. Given the popularity of cinema relative to other cultural forms, there can be little doubt that supporting the emergence of an effective cinematherapy by doing so would be a socially valuable outcome.

## Conclusion

My aim in this study was to show that abstraction provides a useful metric for grouping together observations about how culture and mental health might interact. Necessarily, this is a speculative undertaking from which no definitive conclusions can be expected—which is as well, because no definitive conclusions have emerged. However, what will hopefully be in evidence is that mid-level measures like abstraction, in virtue of how they shape information-bearing structures, can be used to bridge the gap between mental illnesses that themselves actively shape the processing of information.106 If this is allowed, how might such a research programme be taken forward?

The first issue that would need to be addressed centres on the question of content. In the interests of exposition, I have set to one side all considerations of how subject matter might impact on the reception of abstract and concrete modes of representation—despite the obvious fact that content (rather than form) is often the primary driver of response. The problem this poses is how content can be made susceptible to systematic hypothesis testing. One solution might come from dimensional accounts of emotion like the valence-arousal-dominance (VAD) model, which maps variation between emotions into variations in the degree to which each of the three components are associated with a stimulus.107 Though experimentally investigating a system that has four parameters of variation is a formidable task, it is not intractable, and will certainly nuance the abstraction-derived model explored here. Moreover, there are now available large corpuses of words and images that have already assessed for how to trigger the VAD components, which makes the creation of experimental materials substantially easier.108

A second issue comes with the parallel problem of interpersonal variation. Several studies show that a preference for abstraction is either a driver or concomitant of stable personality traits.109 What remains unclear is how this preference relates to anxiety, depression and CLT effects. It may be, for instance, that it merely moderates susceptibility to CLT effects, and can thus be controlled for experimentally. But it may also be that it is implicated in proneness to anxiety or depression in the first place, which makes for a more complicated model. Either way, this interaction needs further research before a complete picture can emerge.

Finally, there is the issue of how the cognitive effects proposed here might be instantiated in the brain. In this regard, recent work on predictive processing and the free energy principle shows that entropy plays a crucial role in how organisms optimise for error reduction in their interaction with the world.110 The crucial idea is that organisms minimise ‘surprisal’—expressed as the log probability of a given stimulus—by either updating their model or probing the environment to gain better data. However, tolerance for surprise is not fixed, and is likely to be mediated by the predictive actions of the dopaminergic and serotonergic systems.111 As these systems are also implicated in mood disorders,112 this opens a potential avenue of connection between CLT-style cultural effects (as mediated by entropy) and the neurotransmitters involved in the regulation of mood. Though I can do no more than point to this possibility here, recent work by Carhart-Harris *et al* on the ‘entropic brain’ model and the impact of serotonin on active and passive coping suggests how it might be pursued by linking the surprisal of a cultural stimulus (relative to background cultural entropy) to the differential activation of 5-HTA1 and 5-HTA2 serotonin receptors.113

I do pretend that these considerations are enough to defend the present survey against its limitations. But they may point to solutions that obviate the need to reject the abstraction hypothesis merely because it is difficult to assess. With respect to the hypothesis itself, only further testing can establish whether it has value. Though such testing with cultural materials will always be difficult, I submit that the potential gains—therapeutic and intellectual—make it a practice that will richly reward being pursued.

## Notes

1. Edward B Tylor (1873), *Primitive Culture: Researches into the Development of Mythology, Philosophy, Religion, Language, Art, and Custom*; Leon J Goldstein (1957), “On Defining Culture”; John Baldwin, Sandra Faulkner, and Michael Hecht (2005), “A Moving Target: The Illusive Definition of Culture.”

2. David Pilgrim, Ann. McCranie, and Recovery (2013), *Recovery and Mental Health: A Critical Sociological Account*; Whitehead et al., *Edinburgh Companion to Crit. Med. Humanit*.; Michel Foucault and Madness (1967), *Madness and Civilization: A History of Insanity in the Age of Reason*.

3. Ana Mari Cauce et al. (2002), “Cultural and Contextual Influences in Mental Health Help Seeking: A Focus on Ethnic Minority Youth”; Giyeon Kim et al. (2013), “Regional Variation of Racial Disparities in Mental Health Service Use Among Older Adults”; Kate Loewenthal (2006), *Religion, Culture and Mental Health*.

4. Yaacov Trope and Nira Liberman (2010), “Construal-Level Theory of Psychological Distance”; Nira Liberman and Yaacov Trope (2008), “The Psychology of Transcending the Here and How.”

5. Tal Eyal and Nira Liberman (2012), “Morality and Psychological Distance: A Construal Level Theory Perspective”; Stephan, Liberman, and Trope, “Politeness and Psychological Distance: A Construal Level Perspective.”; Liberman and Trope, “The Role of Feasibility and Desirability Considerations in near and Distant Future Decisions: A Test of Temporal Construal Theory”; Mueller, Wakslak, and Krishnan, “Construing Creativity: The How and Why of Recognizing Creative Ideas.”

6. Matthew Ratcliffe (2015), *Experiences of Depression: A Study in Phenomenology*, 79.

7. Thomas Fuchs (2013), “Depression, Intercorporeality, and Interaffectivity,” 229.

8. Daniel Smith (2012), *Monkey Mind: A Memoir of Anxiety*, 174.

9. Scott Stossel (2014), *My Age of Anxiety*, 56.

10. Yaacov Trope and Nira Liberman (2010), “Construal-Level Theory of Psychological Distance,” 442.

11. Nira Liberman and Yaacov Trope (1998), “The Role of Feasibility and Desirability Considerations in near and Distant Future Decisions: A Test of Temporal Construal Theory.”

12. Kentaro Fujita et al. (2006), “Spatial Distance and Mental Construal of Social Events.”

13. Cheryl J Wakslak et al. (2006), “Seeing the Forest When Entry Is Unlikely: Probability and the Mental Representation of Events.”

14. Ido Liviatan, Yaacov Trope, and Nira Liberman (2008), “Interpersonal Similarity as a Social Distance Dimension: Implications for Perception of Others’ Actions.”

15. Tal Eyal, Nira Liberman, and Yaacov Trope (2008), “Judging near and Distant Virtue and Vice.”

16. Leaf Van Boven et al. (2010), “Feeling Close: Emotional Intensity Reduces Perceived Psychological Distance.”

17. Joshua Ian Davis, James J Gross, and Kevin N Ochsner (2011), “Psychological Distance and Emotional Experience: What You See Is What You Get”; P. Sol Hart, Richard C. Stedman, and Katherine A. McComas (2015), “How the Physical Proximity of Climate Mitigation Projects Influences the Relationship between Affect and Public Support.”

18. Alison Ledgerwood and Shannon P Callahan (2012), “The Social Side of Abstraction: Psychological Distance Enhances Conformity to Group Norms.”

19. Antonio L. Freitas et al. (2009), “Action-Construal Levels and Perceived Conflict among Ongoing Goals: Implications for Positive Affect.”

20. Adam L Alter, Daniel M Oppenheimer, and Jeffrey C Zemla (2010), “Missing the Trees for the Forest: A Construal Level Account of the Illusion of Explanatory Depth.”

21. Maayan Katzir and Tal Eyal (2013), “When Stepping Outside the Self Is Not Enough: A Self-Distanced Perspective Reduces the Experience of Basic but Not of Self-Conscious Emotions.”

22. Jennifer S. Mueller, Cheryl J. Wakslak, and Viswanathan Krishnan (2014), “Construing Creativity: The How and Why of Recognizing Creative Ideas.”

23. Elena Stephan et al. (2010), “Politeness and Psychological Distance: A Construal Level Perspective.”

24. Caglar Irmak, Cheryl J. Wakslak, and Yaacov Trope (2013), “Selling the Forest, Buying the Trees: The Effect of Construal Level on Seller-Buyer Price Discrepancy.”

25. Katie A McLaughlin and Susan Nolen-Hoeksema (2011), “Rumination as a Transdiagnostic Factor in Depression and Anxiety”; Jennifer A. Harrington and Virginia Blankenship (2002), “Ruminative Thoughts and Their Relation to Depression and Anxiety.”

26. American Psychiatric Association (2013b), *Diagnostic and Statistical Manual of Mental Disorders*, 160–61.

27. American Psychiatric Association (2013b), 123.

28. E Watkins and J. D. Teasdale (2001), “Rumination and Overgeneral Memory in Depression: Effects of Self-Focus and Analytic Thinking.” 353.

29. Edward Watkins (2011), “Dysregulation in Level of Goal and Action Identification across Psychological Disorders” 270.

30. Matthew Ratcliffe (2015), 10.

31. Matthew Ratcliffe (2015), 31.

32. Matthew Ratcliffe (2015), 79.

33. Matthew Ratcliffe (2015), 71.

34. Matthew Ratcliffe (2015), 67.

35. Matthew Ratcliffe (2015), 15.

36. American Psychiatric Association (2013b), *Diagnostic and Statistical Manual of Mental Disorders*, 222.

37. Edward Watkins (2011), “Dysregulation in Level of Goal and Action Identification across Psychological Disorders” 271.

38. E Watkins and J. D. Teasdale (2001), “Rumination and Overgeneral Memory in Depression: Effects of Self-Focus and Analytic Thinking.”

39. A Mathews (1990), “Why Worry? The Cognitive Function of Anxiety” 456.

40. Allison J Ouimet (2009), “Cognitive Vulnerability to Anxiety: A Review and an Integrative Model” 460; Yair Bar-Haim, Dominique Lamy, and Lee Pergamin (2007), “Threat-Related Attentional Bias in Anxious and Nonanxious Individuals: A Meta-Analytic Study”; Merel Kindt and Marcel Van Den Hout (2001), “Selective Attention and Anxiety: A Perspective on Developmental Issues and the Causal Status”; Karin Mogg and Brendan P Bradley (2004), “A Cognitive-Motivational Perspective on the Processing of Threat Information and Anxiety.”

41. Reddit (2015), “Psychiatrists/Psychologists of Reddit, What Is the Most Profound or Insightful Thing You Have Ever Heard from a Patient with a Mental Illness? : AskReddit.”

42. Reddit.

43. Reddit.

44. Eileen O’Meara (2017), “Panic Attack!”

45. Meredith Arthur (2017), “GAD: Hard to Recognize | Anxiety and Depression Association of America, ADAA.”

46. Ernst H W Koster, Elaine Fox, and Colin MacLeod (2009), “Introduction to the Special Section on Cognitive Bias Modification in Emotional Disorders.”

47. Aliza Werner-Seidler and Michelle L Moulds (2012), “Mood Repair and Processing Mode in Depression.”

48. Aliza Werner-Seidler and Michelle L Moulds (2012), 476.

49. E. R. Watkins et al. (2012), "Guided self-help concreteness training as an intervention for major depression in primary care: a Phase II randomized controlled trial," 222.

50. Nader Amir et al. (2009), “Attention Modification Program in Individuals with Generalized Anxiety Disorder.”

51. Nader Amir and Charles T Taylor (2012), “Combining Computerized Home-Based Treatments for Generalized Anxiety Disorder: An Attention Modification Program and Cognitive Behavioral Therapy”; Yuko Hakamata et al. (2010), “Attention Bias Modification Treatment: A Meta-Analysis towards the Establishment of Novel Treatment for Anxiety”; Han-Joo Lee et al. (2015), “Computerized Attention Retraining for Individuals with Elevated Health Anxiety.”

52. Stefan G Hofmann (2008), “Cognitive Processes during Fear Acquisition and Extinction in Animals and Humans: Implications for Exposure Therapy of Anxiety Disorders”; Mark B Powers and Paul M G Emmelkamp (2008), “Virtual Reality Exposure Therapy for Anxiety Disorders: A Meta-Analysis.”

53. Tom J Barry, Bram Vervliet, and Dirk Hermans (2015), “An Integrative Review of Attention Biases and Their Contribution to Treatment for Anxiety Disorders” 9.

54. Wilhelm Worringer (1997), *Abstraction and Empathy: A Contribution to the Psychology of Style*, 4.

55. Wilhelm Worringer (1997), 15.

56. Wilhelm Worringer (1997), 16.

57. Clifford Geertz (1973), *The Interpretation of Cultures: Selected Essays*; Hans-Georg Gadamer (2004), *Truth and Method*; Paul Ricoeur (2003), *The Rule of Metaphor*.

58. Pascal Boyer (2001), *Religion Explained: The Evolutionary Origins of Religious Thought*; James Carney and Pádraig Mac Carron (2017), “Comic-Book Superheroes and Prosocial Agency: A Large-Scale Quantitative Analysis of the Effects of Cognitive Factors on Popular Representations”; Tamás Dávid-Barrett and James Carney (2015), “The Deification of Historical Figures and the Emergence of Priesthoods as a Solution to a Network Coordination Problem”; David Herman (2013), “Narrative and Mind: Directions for Inquiry”; Emily T Troscianko (2013), “Cognitive Realism and Memory in Proust’s Madeleine Episode”; Lisa Zunshine (2012), *Getting Inside Your Head: What Cognitive Science Can Tell Us About Popular Culture*.

59. P Matthijs Bal and Martijn Veltkamp (2013), How Does Fiction Reading Influence Empathy? An Experimental Investigation on the Role of Emotional Transportation”; David Comer Kidd and Emanuele Castano (2013), “Reading Literary Fiction Improves Theory of Mind”; James Carney, Rafael Wlodarski, and Robin Dunbar (2014), “Inference or Enaction? The Impact of Genre on the Narrative Processing of Other Minds”; Carney and Robertson, “People Searching for Meaning in Their Lives Find Literature More Engaging.”

60. James Carney (2017), “The Space Between Your Ears: Construal Level Theory, Cognitive Science and Science Fiction.”

61. Claude E Shannon (1948), “A Mathematical Theory of Communication”; David McKay and Information Theory (2003), *Information Theory, Inference, and Learning Algorithms*; C. E. Shannon (1951), “Prediction and Entropy of Printed English.”

62. Alexander Leonidovich Kuzemsky (2017) , *Statistical Mechanics and the Physics of Many-Particle Model Systems*, 275.

63. David G Edwards (2014), *Art Therapy*.

64. Anthony Vidler (2001), *Warped Space: Art, Architecture, and Anxiety in Modern Culture*, 1.

65. Anthony Vidler (2001), 1.

66. Christopher S Henshilwood, Christopher S et al. (2002), “Emergence of Modern Human Behavior: Middle Stone Age Engravings from South Africa.”

67. Paul G Bahn (2012) , *Cave Art: A Guide to the Decorated Ice Age Caves of Europe*.

68. D.W Harding (2007) , *The Archaeology of Celtic Art*; Barry W Cunliffe (1997), *The Ancient Celts*.

69. Nancy Curry and Tim Kasser (2005), “Coloring To Reduce Anxiety.”

70. Renée van der Vennet and Susan Serice (2012), “Can Coloring Mandalas Reduce Anxiety? A Replication Study.”

71. David Alan Sandmire et al. (2012), “The Influence of Art Making on Anxiety: A Pilot Study.”

72. H Wadeson (1971), “Characteristics of Art Expression in Depression”; Harriet Wadeson (2010), *Art Psychotherapy*.

73. Gil Bar-Sela et al. (2007), “Art Therapy Improved Depression and Influenced Fatigue Levels in Cancer Patients on Chemotherapy.”

74. Sarah C. Slayton, Jeanne D'Archer, and Frances Kaplan (2010), “Outcome Studies on the Efficacy of Art Therapy: A Review of Findings”; Matthew W. Reynolds, Laura Nabors, and Anne Quinlan (2000), “The Effectiveness of Art Therapy: Does It Work?”

75. Jonathan J Detrixhe (2010), “Souls in Jeopardy: Questions and Innovations for Bibliotherapy with Fiction”; M L Lanza and Marilyn Lewis Lanza (1991), “Literature: A Vehicle for Emotional Connection between Clinician and Client”; Gavin Francis (2017), “Storyhealing”; Pehrsson, Dale-Elizabeth Pehrsson (2015) “Fictive Bibliotherapy and Therapeutic Storytelling with Children Who Hurt”; Josie Billington (2016), *Is Literature Healthy?*

76. Jonathan Bate and Andrew Schuman (2016), “Books Do Furnish a Mind: The Art and Science of Bibliotherapy”, 743.

77. Paul Montgomery and Kathryn Maunders (2015), “The Effectiveness of Creative Bibliotherapy for Internalizing, Externalizing, and Prosocial Behaviors in Children: A Systematic Review.”

78. Keith Oatley (1995), “A Taxonomy of the Emotions of Literary Response and a Theory of Identification in Fictional Narrative”; Keith Oatley (1999), “Meetings of Minds: Dialogue, Sympathy, and Identification, in Reading Fiction.”

79. American Psychiatric Association (2013a), *Diagnostic and Statistical Manual of Mental Disorders, Fifth Edition*.

80. Monika Fludernik (2004), *Towards a “Natural” Narratology*; Gerald Prince (2004), “Revisiting Narrativity.”

81. David Herman (2002), *Story Logic: Problems and Possibilities of Narrative*, 103.

82. Alan Dundes (1964) , *The Morphology of North American Indian Folktales*; Claude Lévi-Strauss (1964), *Mythologiques, Vols. I–IV*; Christian Metz (1991), *Film Language: A Semiotics of the Cinema*; Vladimir Propp (1968), *Morphology of the Folktale*.

83. David Herman (2011), *Basic Elements of Narrative*; A. J. Greimas and Catherine Porter (1977), “Elements of a Narrative Grammar”; H Porter Abbott (2000), “The of Evolutionary Origins of the Storied Mind: Modeling the Prehistory of Narrative Consciousness and Its Discontents.”

84. A.J Greimas (1983), *Structural Semantics: An Attempt at a Method*; Greimas, “Elements of a Narrative Grammar”; A.J Greimas (1987), “Actants, Actors, and Figures.”

85. A.J Greimas (1983), *Structural Semantics: An Attempt at a Method*, 200.

86. Dorrit Cohn (1978), *Transparent Minds: Narrative Modes for Presenting Consciousness in Fiction*, 9.

87. Chris D Frith and Uta Frith (2007), “Social Cognition in Humans.”

88. James Joyce (1939), *Finnegans Wake*.

89. Virginia Woolf (1931), *The Waves*.

90. Arthur N Applebee (1989), *The Child’s Concept of Story: Ages Two to Seventeen*.

91. Samuel Beckett (1958), *The Unnamable*, 1.

92. James Carney and Cole Robertson (2018), “People Searching for Meaning in Their Lives Find Literature More Engaging.”

93. Calla E. Y. Glavin and Paul Montgomery (2017), “Creative Bibliotherapy for Post-Traumatic Stress Disorder (PTSD): A Systematic Review.”

94. Paul Montgomery and Kathryn Maunders (2015), “The Effectiveness of Creative Bibliotherapy for Internalizing, Externalizing, and Prosocial Behaviors in Children: A Systematic Review.”

95. Emily T Troscianko (2018), “Literary Reading and Eating Disorders: Survey Evidence of Therapeutic Help and Harm.”

96. Michael Lee Powell and Rebecca A. Newgent (2010), “Improving the Empirical Credibility of Cinematherapy,” 41.

97. E Katz (1947), “Audio-Visual Aids for Mental Hygiene and Psychiatry”; E Katz (1945), “A Social Therapy Program for Neuropsychiatry in a General Hospital.”

98. Shannon B. Dermer and Jennifer B. Hutchings (2000), “Utilizing Movies in Family Therapy: Applications for Individuals, Couples, and Families”; Carla Gramaglia et al. (2011), “Cinematherapy in the Day Hospital Treatment of Patients with Eating Disorders. Case Study and Clinical Considerations.”

99. Linda Berg-Cross, Pamela Jennings, and Rhoda Baruch (1990), “Cinematherapy: Theory and Application”; Emily Marsick (2010), “Cinematherapy with Preadolescents Experiencing Parental Divorce: A Collective Case Study.”

100. Vitor Hugo Sambati Oliva, Andréa Vianna, and Francisco Lotufo Neto (2010), “Cinematherapy as Psychotherapeutic Intervention: Characteristics, Applications and Identification of Cognitive-Behavior Techniques.”

101. Fritz Heider et al. (1944), “An Experimental Study of Apparent Behavior.”

102. Francis Ford Coppola (1979), *Apocalypse Now*.

103. Tamás Dávid-Barrett and Robin Dunbar (2013), “Processing Power Limits Social Group Size: Computational Evidence for the Cognitive Costs of Sociality.”

104. D. E Berlyne (1970), “Novelty, Complexity, and Hedonic Value.”

105. Richard Dyer (2004), *Heavenly Bodies: Film Stars and Society*, 5.

106. A. K Wittenborn et al. (2016), “Depression as a Systemic Syndrome: Mapping the Feedback Loops of Major Depressive Disorder”; Clark, *Surfing Uncertainty: Prediction, Action, and the Embodied Mind*.

107. Albert Mehrabian (1980) , *Basic Dimensions for a General Psychological Theory: Implications for Personality, Social, Environmental, and Developmental Studies*.

108. Peter Lang, Margaret Bradley, and B N Cuthbert (2008), “International Affective Picture System (IAPS): Affective Ratings of Pictures and Instruction Manual”; Artur Marchewka et al. (2014), “The Nencki Affective Picture System (NAPS): Introduction to a Novel, Standardized, Wide-Range, High-Quality, Realistic Picture Database”; Amy Beth Warriner, Victor Kuperman, and Marc Brysbaert (2013), “Norms of Valence, Arousal, and Dominance for 13,915 English Lemmas.”

109. Tomas Chamorro-Premuzic and Adrian Furnham (2004), “Art Judgment: A Measure Related to Both Personality and Intelligence?”; Adrian Furnham and John Walker (2001), “Personality and Judgements of Abstract, Pop Art, and Representational Paintings”; H. J. Eyesenck and Hans Jürgen Eysenck (1940), “The General Factor in Aesthetic Judgments.”

110. Andy Clark (2016), *Surfing Uncertainty: Prediction, Action, and the Embodied Mind*; Karl Friston (2010), “The Free-Energy Principle: A Unified Brain Theory?”; Karl J Friston, Jean Daunizeau, and Stefan J Kiebel (2009), “Reinforcement Learning or Active Inference?”; Mateus Joffily and Giorgio Coricelli (2013), “Emotional Valence and the Free-Energy Principle.”

111. Sara Matias et al. (2017), “Activity Patterns of Serotonin Neurons Underlying Cognitive Flexibility”; Friston et al., “Dopamine, Affordance and Active Inference.”

112. D Baldwin and S Rudge, 1995, “The Role of Serotonin in Depression and Anxiety.”

113. R L Carhart-Harris and D J Nutt (2017), “Serotonin and Brain Function: A Tale of Two Receptors”; Robin L Carhart-Harris et al. (2014), “The Entropic Brain: A Theory of Conscious States Informed by Neuroimaging Research with Psychedelic Drugs.”
